# Survival capabilities of *Escherichia coli* O26 isolated from cattle and clinical sources in Australia to disinfectants, acids and antimicrobials

**DOI:** 10.1186/s12866-017-0963-0

**Published:** 2017-03-01

**Authors:** Salma A. Lajhar, Jeremy Brownlie, Robert Barlow

**Affiliations:** 1 School of Natural Sciences, Griffith University, Brisbane, QLD Australia; 2 CSIRO Agriculture and Food, Brisbane, QLD Australia; 3 Present address: CSIRO Agriculture and Food, 39 Kessels Rd, Coopers Plains, QLD 4108 Australia

**Keywords:** *E. coli* O26, Virulence marker, Pathotype, Antimicrobial agent, Disinfectant, Organic acid

## Abstract

**Background:**

After *E. coli* O157, *E. coli* O26 is the second most prevalent enterohaemorrhagic *E. coli* (EHEC) serotype identified in cases of foodborne illness in Australia and throughout the world. *E. coli* O26 associated foodborne outbreaks have drawn attention to the survival capabilities of this organism in a range of environments. The aim of the present study was to assess the ability of *E. coli* O26 to survive the effects of disinfectants, acids and antimicrobials and investigate the possible influence of virulence genes in survival and persistence of *E. coli* O26 from human and cattle sources from Australia.

**Results:**

Initial characterization indicated that *E. coli* O26 are a genetically diverse group that were shown to belong to a number of pathotypes. Overall, 86.4% of isolates were susceptible to all antimicrobials tested with no significant differences in resistance observed between pathotypes. A representative subset of isolates (*n* = 40) were selected to determine their ability to survive disinfectants at proposed industry working concentrations and acid stress. Profoam, Kwiksan 22, and Topactive DES. were able to inhibit the growth of 100% of isolates. The remaining three disinfectants (Dairy Chlor 12.5%, Envirosan and Maxifoam) were not effective against the subset of 40 *E. coli* O26. Finally, elevated MICs (1,024 to 4,096 μg/ml) of acetic, propionic, lactic, and citric acids were determined for the majority of the isolates (85%).

**Conclusions:**

Australian *E. coli* O26 isolates belong to a range of pathotypes that harbor differing virulence markers. Despite this, their response to antimicrobials, disinfectants and acids is similar confirming that stress response appears unrelated to the presence of EHEC virulence markers. Notwithstanding, the tolerance to disinfectants and the elevated acid MICs for EHEC and the other *E. coli* O26 pathotypes examined in this study may contribute to bacterial colonization on food contact surfaces and subsequent foodborne illness caused by this pathogen.

## Background

Enterohaemorrhagic *Escherichia coli* (EHEC) strains have been associated with a number of food-borne outbreaks which have led to life threatening sequelae such as hemolytic uremic-syndrome (HUS) and hemolytic colitis (HC) [1–3]. Epidemiological surveillance indicates that *E. coli* of O157:H7 serotype is the most frequently encountered EHEC implicated in sporadic and outbreak cases of illness [4]. However, other non-O157 serotypes such as O26:H11, O45:H2, O103:H2, O111:H8, O121:H19, O145:H28 and their non-motile forms have emerged and are now considered an important cause of human infection resulting in HUS [5]. Among the non-O157 serotypes, *E. coli* O26:H11 is one of the major serotypes of concern [3, 6].

Although it is not completely understood which suite of bacterial virulence determinants are most necessary for *E. coli* O26 to cause disease in humans, isolates recovered from human clinical samples typically possess Shiga toxins 1 or 2 or both (*stx*_*1*_, *stx*_*2*_) encoded by lambdoid bacteriophages, the *E. coli* attaching and effacing gene (*eae*) which is located on the LEE island and is necessary for bacterial colonisation of the gut and formation of the A/E lesion, and enterohemolysin *(ehx*), a plasmid encoded virulence factor thought to work synergistically with *stx* and contribute to the pathogenicity of EHEC [7–10]. It has been recognised that cattle represent a major reservoir of *E. coli* O26 [11–13]. Exposure to this pathogen can occur by a variety of routes including contact with animals on farms, consumption of contaminated meat, milk and its derivatives, water, spinach, sliced watermelon, clover sprouts, blueberries and strawberries [11, 14–20]. The contamination of food products with either *stx* positive or *stx* negative *E. coli* O26 strains has been reported previously [21–25] and in some cases it has resulted in the recall of food products and a number of outbreaks [6, 18, 26]. In 2005, there was an outbreak in France due to consumption of unpasteurised cow cheese contaminated with EHEC O26 [27]. In 2007, EHEC O26 infections occurred amongst consumers of ice cream produced from pasteurised milk made and sold at a farm in Belgium [23]. Additionally, multistate outbreaks of EHEC O26 infections in the USA have been also reported by the CDC in 2010, 2011, 2013 and 2015 [16, 17, 28].

The detection of *E. coli* O26 isolates in various environments including food, processing equipment and food contact surfaces and identifying them as a causative agent for a number of food-borne outbreaks creates the need for implementing prevention strategies to control this pathogen. Food producers and processors can use a range of antimicrobial agents such as sanitizers and disinfectants to assist in controlling this organism. Resistance of *E. coli* strains recovered from various environments to a variety of antimicrobial agents has been reported [29–32]. Variations in the response of different strains of *E. coli* to a range of disinfectants was demonstrated by Skaloud et al., [31] and Beier et al., [32] and suggest that the stress response of a range of *E. coli* strains may be highly variable. To date, most studies have focused on the survival capability of *E. coli* O157:H7 and minimal information is known about the response of *E. coli* O26 strains to these types of stressors. It cannot be assumed that *E. coli* O26 will respond similarly to *E. coli* O157:H7 or other *E. coli* strains when exposed to antimicrobial agents. We hypothesised that EHEC O26 have enhanced ability to persist and survive antimicrobial intervention in their planktonic state and that this consequently contributes to EHEC incidence and infection. Therefore, the aim of the present study was to assess the ability of *E. coli* O26 recovered from human clinical cases and cattle sources to resist the effects of disinfectants, acids and antimicrobials in the planktonic state and investigate the possible association of virulence genes such as *stx* and *eae* with the survival of Australian *E. coli* O26 from human and cattle sources.

## Methods

### Bacterial strains

A total of 88 *E. coli* O26 isolates collected previously from human clinical (10) and cattle (78) sources between 1995 and 2013 were utilised in this study. *E. coli* O26 isolates were initially selected with broad criteria based on virulence profiles, time and source. Isolates stored at -80°C in protect bacterial preservers (Technical Service Consultants Ltd) were subcultured on tryptone soya agar (TSA; Oxoid, UK) and incubated at 37 °C overnight.

### PCR

Whole cell suspensions were created by suspending a single colony in 200 μl sterile Milli-Q water and tested by PCR for the presence of *stx* (*stx*_1_ and *stx*_2_), *eae*, *ehx*, *eae* conserved fragment (*ecf*), and bundle forming pilus *(bfpA*)*,* using the primers shown in Table 1. PCR master mix containing 10X Dream Taq™ Buffer (Thermo Fisher Scientific, Australia), 250 mM dNTPs (Thermo Fisher Scientific, Australia), 0.02 mg/ml bovine serum Albumin (Sigma-Aldrich, USA), 12.5 pmol forward and reverse primer (GeneWorks, Australia) and 1.25 U Taq DNA polymerase (GeneWorks, Australia) was used. PCR products were subjected to gel electrophoresis on 2% agarose gels for 45 min at 100 V with resulting bands then visualised using a UV transilluminator. The anticipated amplicon size for each PCR product is shown in Table 1.

**Table 1:** PCR Primer sequences used in this study

Primers	Sequence	Amplicon size	References
stx1-F	5'-ATAAATCGCCATTCGTTGACTAC-3'	180	[4]
stx1-R	5'-AGAACGCCCACTGAGATCATC-3'		
stx2-F	5'-GGCACTGTCTGAAACTGATCC-3'	255	[4]
stx2-R	5'-TCGCCAGTTATCTGACATTCTG-3'		
eae-F	5'-GACCCGGCACAAGCATAAGC-3'	284	[4]
eae-R	5'-CCACCTGCAGCAACAAGAGG-3'		
hlyA-F	5'-GCATCATCAAGC GTACGT TCC-3'	534	[4]
hlyA-R	5'-AATGAGCCAAGCTGGTTAAGC T-3'		
wzx O26-F	5'-CGCGACGGCAGAGAAAATT-3'	326	[47]
wzx O26-R	5'-ACAATCCAACCGAACCAAAC-3'		This study
ecf-F	5'-TATCAGCACCAAAGAGCGGGAACA-3'	99	[48]
ecf-R	5'-CCCTTATGAAGAGCCAGTACTGAA-3'		
rmlA 30snp-F	5'-AAGTCGCAGGCTTGT-3'	484	This study
rmlA 30snp-R	5'-CGAAGACCCGCTAAC-3'		
BFPA300-F	5'-GGAAGTCAAATTCATGGG-3'	300	[49]
BFPA300-R	5'-GGAATCAGACGCAGACTGGT-3'		

### Detection of rmlA SNP using RFLP

A single nucleotide difference (G→T) at position 30 within *rmlA* has been shown to be associated with the presence of *stx* in *E. coli* isolates [33]. Primers rmlA 30snp-F and rmlA 30snp-R were used to amplify a 484 bp portion of *rml*A (Table 1). Amplified PCR products were digested for 4 h at 37 °C using the restriction enzyme *Aci*I. PCR products were subjected to gel electrophoresis on 2% agarose gels for 45 min at 100 V with resulting bands then visualised using a UV transilluminator. Isolates harbouring the *rml*A SNP yielded 10 bp (not visible), 161 bp and 313 bp fragments whereas those isolates without the SNP yielded 10 bp (not visible), 109, 161 and 204 bp fragments.

### Antimicrobial Resistance (AMR)

To determine the AMR phenotype of *E. coli* O26 isolates a custom susceptibility panel AUSVN2 (TREK Diagnostics, UK) designed specifically for testing Australian Gram-negative isolates was used. All plates were inoculated and assessed using the Sensititre system (TREK Diagnostics). Antimicrobials that were tested are cefazolin, cefotaxime, ceftiofur, amoxicillin / clavulanic acid, nalidixic acid, cefoxitin, ciprofloxacin, meropenem, ceftriaxone, gentamicin, ampicillin, trimethoprim / sulfamethoxazole, chloramphenicol, kanamycin, tetracycline and streptomycin. The Clinical and Laboratory Standards Institute (CLSI) criteria were utilised to identify antimicrobial resistance breakpoints when available; otherwise European Committee on Antimicrobial Susceptibility Testing (EUCAST) and National Antimicrobial Resistance Monitoring System (NARMS) values were used. *E. coli* ATCC 25922 was used as a control strain.

### Pulse-field gel electrophoresis (PFGE)

PFGE was performed using the standardised PulseNet protocol with chromosomal DNA of *Salmonella ser. Braenderup* H9812 digested with XbaI (Roche diagnostics, USA) used as a molecular size marker [34, 35]. PFGE gels were analysed using BioNumerics V7.5 (Applied Maths, Belgium).

### Disinfectant and acid susceptibility

A total of six disinfectants and four acids were evaluated for their antimicrobial efficacy against *E. coli* O26 isolates. The disinfectants and acids tested were Topactive DES (Ecolab Pty Ltd, Australia), Dairy Chlor 12.5% (Campbell Cleantec, Australia), Maxifoam (Ecolab Pty Ltd, Australia), Envirosan (Ecolab Pty Ltd, Australia), Profoam (Jasol Australia, Australia), Kwiksan 22 (Ecolab Pty Ltd, Australia), acetic acid (Sigma-Aldrich, Mexico), citric acid (Sigma-Aldrich, Japan), lactic acid (Sigma-Aldrich, Japan), and propionic acid (Sigma-Aldrich, Japan). Disinfectants tested in this study are approved for use in Australian food industries and food processing areas. Each disinfectant was tested at concentrations spanning the recommended working concentrations. The active components of each of the disinfectants are as follows: Topactive Des.: hydrogen peroxide solution (<10%), acetic acid (<10%), amines, C12-16- alkyldimethyl (<10%), N-oxides (<10%), peracetic acid (<10%); ﻿Dairy Chlor: sodium hypochlorite (10- < 30%), sodium hydroxide (<10%); Maxifoam: potassium hydroxide (<10%), builder (<10%), alkaline salts (<10%), anionic surfactant (<10%), surfactants (<10%), hydrotrope (<10%), sodium hypochlorite (<10%) and scale inhibitors (<10%); Envirosan: dodecylbenzenesulfonic acid (<10%), propanoic acid, 2-hydroxy-,(s) (<10%); Profoam: quaternary ammonium compounds (0-5%), surfactants (10-30%); Kwiksan 22: quaternary ammonium compounds, benzyl-C8-18-alkyldimethyl, chlorides (10- < 30%). Evaluation of the effectiveness of the antimicrobial activities of disinfectants and acids and subsequent determination of the susceptibility profiles were performed on polystyrene microtiter plate using broth microdilution method as described previously [36, 37]. Briefly, a single colony from each isolate was streaked onto TSA agar plates and grown for 18 to 24 h at 37 °C. Working solutions for each disinfectant were prepared by diluting with Müller-Hinton broth (MHB; Oxoid, UK) then sterilising using a 0.45 μm syringe filtre (Sartorius Stedim Biotech GmbH, Germany). The working solutions were then two-fold serially diluted to achieve the test concentrations and pH for each concentration was determined. The disinfectant and acid ranges tested were: Topactive DES. (0.0156%-16%), Dairy Chlor 12.5% (0.0188%-12.5%), Maxifoam (0.025%-25.6%), Envirosan (0.025%-25.6%), Profoam: 0.078%-8%, Kwiksan 22 (0.0035%-3.6%), acetic acid (64-65,536 μg/ml), lactic acid (64-65,536 μg/ml), citric acid: (64-65,536 μg/ml) and propionic acid (64-65,536 μg/ml). *E. coli* O157:H7 Sakai strain was used as a control for the survival of isolates to disinfectant challenge and acid challenge assay.

## Results

### Characterization of *E. coli* O26

A total of 88 isolates were screened for the presence of PCR gene targets: *stx﻿*_*1*_, *stx*_*2*_, *eae*, *ehx*, *ecf*, *bfp* and the *rml*A SNP. A summary of the PCR screening and characterization of clinical and cattle isolates is shown in Table 2. All of the 88 isolates were found to be negative for *stx﻿*_*2*_ and *bfp.* Based on the results of the PCRs four distinct groupings were formed and subsequently referred to here as pathotypes: EHEC, potential EHEC (pEHEC), atypical Enteropathogenic *E. coli* (aEPEC) and Non-toxigenic *E. coli* (NTEC). The EHEC group includes all human clinical and 40 (51.2%) cattle isolates. Of the remaining cattle isolates, 33 (42.3%) were aEPEC, three (3.8%) were pEHEC, and two (2.6%) were NTEC.

**Table 2:** Prevalence of genetic markers in *E. coli* O26 from clinical and cattle sources

Pathotypes	Virulence makers	No of isolates	Source
EHEC	*stx* (*stx﻿*_1_), *eae*, *ehx*, *ecf,* SNP within *rml*A	50	Clinical and cattle
pEHEC	*eae*, *ehx*, *ecf,* SNP within *rml*A	3	Cattle
aEPEC	*eae*	33	Cattle
NTEC	Negative for all virulence markers tested	2	Cattle

### Antimicrobial Resistance (AMR)

A total of 88 isolates were assessed for their resistance to 17 antimicrobials. The distribution of minimum inhibitory concentrations (MICs) for each antimicrobial, concentrations tested and resistance breakpoints are presented in Table 3. Overall, there was a low level of resistance among the cattle isolates with 86.4% of all isolates susceptible to all antibiotics tested in this study. In total, 12 (13.6%) *E. coli* O26 isolates (10 EHEC and two aEPEC) exhibited resistance to at least one antimicrobial. Of the 12 isolates, four were resistant to only one antimicrobial with resistance to streptomycin or nalidixic acid observed in two and one EHEC isolates from cattle, respectively, and a single aEPEC isolate from cattle demonstrating resistance to tetracycline. Multidrug resistance was observed in three human clinical and five cattle isolates. The most common co-resistance phenotype observed was ampicillin-kanamycin-streptomycin-tetracycline (one cattle EHEC isolate and two human clinical EHEC isolates) and ampicillin-streptomycin (three cattle EHEC isolates) while resistance to both chloramphenicol-streptomycin and ampicillin-streptomycin-tetracycline were found in a human clinical EHEC isolate and a cattle aEPEC isolate, respectively. Although AMR appeared largely constrained to EHEC isolates with 20% (10/50) demonstrating resistance to at least one antimicrobial, the differences in AMR prevalence between pathotypes was found to not be significant (*p* = 0.05).

**Table 3:**
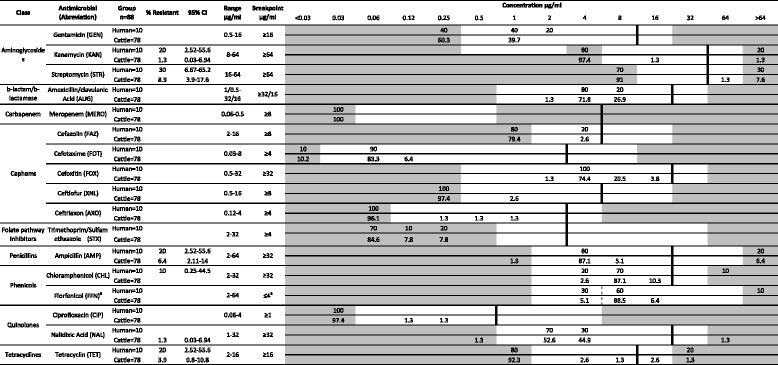
Distribution of antimicrobial MICs among *E. coli* O26 isolates from cattle and human sources

^a^Only a susceptible breakpoint (≤4 μg/ml) has been established. Isolates with an MIC ≥8 μg/ml are reported as non-susceptible. Vertical lines indicate breakpoints for resistance. The white fields indicate the dilution range tested for each antimicrobial. Grey area indicates MIC values greater than or less than the tested concentration. Number of isolates is in percentage (%).^*^CI: Confidence intervals

### PFGE analysis

**Fig. 1:**
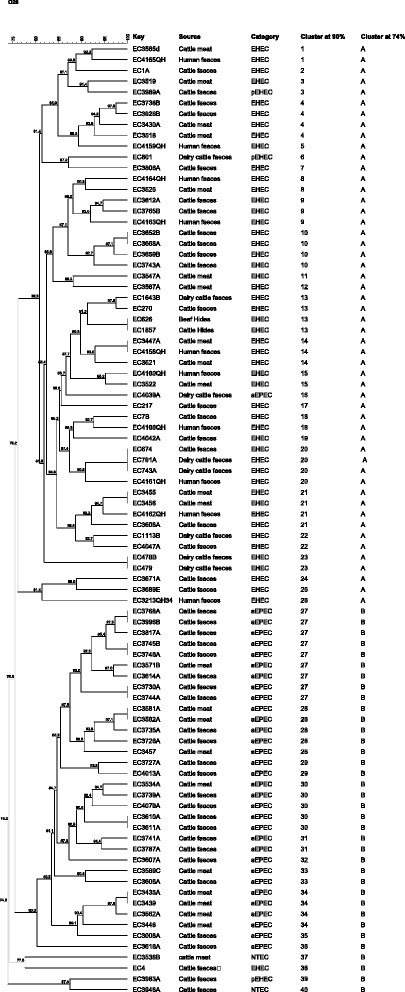
PFGE profiles and clusters of O26 isolates investigated in this study. All 88 isolates were analysed by PFGE with XbaI, and cluster analysis of the patterns was performed by BioNumerics V7.5 software using the Dice coefficient and unweighted pair group method (UPGMA). The degree of similarity (%) is shown on the scale at the top left of the figure. The cut-off level of 90% was chosen to assign isolates to the same cluster. At 74% similarity isolates were assigned to 2 clusters (a & b)

Analysis of PFGE patterns revealed that the *E. coli* O26 strains in this study are highly diverse with similarity between isolates ranging from 71.4-100% (Fig. 1). Comparison of PFGE profiles of 88 isolates identified 75 distinct PFGE patterns at a similarity cut-off level of 100%. At a cut-off value of 90%, isolates could be grouped into 40 clusters, of which, 19 PFGE clusters were represented by a single isolate and the remaining 69 isolates grouped into 21 clusters containing between two and nine isolates. Of the 69 isolates, 44 had distinct PFGE patterns with the remaining 25 isolates splitting into 11 clusters of 2 indistinguishable isolates and 1 cluster of 3 indistinguishable isolates. Of note, two isolates that were unrelated temporally (one from cattle and one from human) produced indistinguishable PFGE patterns. PFGE patterns were classified into two main clusters designated A and B at a similarity level of 74%. Cluster A included 49 (98%) of EHEC, two pEHEC and one aEPEC isolate. Isolates grouped in cluster B were 97% (32/33) aEPEC as well as two NTEC, a single pEHEC and a single EHEC isolate. An association between clusters and AMR isolates were not found (*P* value > 0.05). To capture the diversity of *E. coli* O26 strains, a subset of O26 isolates (*n* = 40) representing various AMR profiles, a range of *E. coli* O26 pathotypes and a diverse PFGE pattern, were then chosen for subsequent tests.

### Susceptibility to disinfectants

The effectiveness of disinfectants currently used in the food industry against 40 *E. coli* O26 was examined in this study and results are shown in Table 4. The proposed industry recommended concentrations for Kwiksan, Profoam, Topactive DES, Dairy Chlor 12.5%, Maxifoam and Envirosan were 0.45, 1, 1, 0.3, 1.6, 0.4%, respectively. The most effective disinfectants with respect to their suggested working concentrations were Kwiksan 22, Topactive DES and Profoam with each able to inhibit the growth of the strains tested with MICs at or below the working concentrations. Dairy Chlor 12.5%, Maxifoam and Envirosan were less effective against the 40 *E. coli* O26 examined in this study with all strains able to grow at a concentration at or above the suggested working concentrations. Importantly, *E. coli* O26 isolates missing any or all EHEC virulence markers (i.e., aEPEC, pEHEC and NTEC) were able to survive the same concentrations of disinfectant tested in our study against EHEC isolates, showing the same MICs_%_ as EHEC. When comparing the effective concentrations of disinfectants required for *E. coli* O26 isolates and the control strain *E. coli* O157:H7 Sakai, similar effectiveness was observed with elevated MICs demonstrated against Dairy Chlor 12.5%, (MIC = 2.4%), Maxifoam (MIC = 3.2%) and Envirosan (MIC = 1.6%). The remaining three disinfectants (Kwiksan 22, Topactive DES. and Profoam) that have shown to be effective against *E. coli* O26 were also effective against *E. coli* O157:H7 Sakai at the proposed industry working concentrations.

**Table 4:**
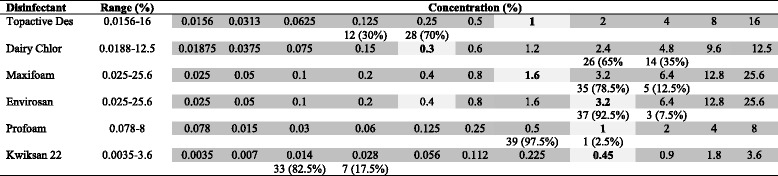
Distribution of disinfectant MICs among 40 *E. coli* O26 isolates from cattle and human sources

Light Grey fields indicate the recommended working concentrations for each disinfectant, Dark Grey fields indicate the dilution range tested for each disinfectant agents. Numbers in the white field indicates number and percentage of isolates susceptible to disinfectants at the tested concentration

### Susceptibility to acids

The MIC distribution profiles of 40 *E. coli* O26 isolates against four acids (acetic, propionic, lactic, and citric acids) are shown in Table 5. For comparison of MICs of *E. coli* O26 to acids with different molecular weights, the values for molar MICs (MICs_molar_) were used. Based on Weight/Volume (w/v) concentrations it appears that the order of acids with the most effect on the *E. coli* O26 strains is acetic acid and propionic then lactic followed by citric acid with MICs of 1,024, 1024, 2,048, and 4,096 μg/ml, respectively (Table 5). Recalculation of the MICs to molar values reveals that propionic, acetic, citric and lactic acids have MICs_molar_ of 13.82, 17.05, 21.3 and 22.7 mmole/ml, respectively, suggesting that propionic acid shows higher efficacy than acetic acid at retarding the growth of *E. coli* O26. When exposed to acids, the MICs for all 40 isolates occurred at an acetic acid pH and propionic acid pH that was much less acidic than that of the other two acids. The MICs_molar_ for 87.5% (35/40) and 12.5% (5/40) of isolates occurred at an acetic acid pH 4.08 and 4.42, respectively. For propionic acid the MICs_molar_ for 100% (40/40) of isolates occurred at pH of 4.55. When exposed to citric acid, the MICs_molar_ for 87.5% (35/40) and 12.5% (5/40) of isolates were observed at citric acid pH of 3.75 and 3.31, respectively, whereas the MICs_molar_ for 100% (40/40) of the isolates occurred at lactic acid pH of 3.67. No significant differences in susceptibility to acids was observed between pathotypes regardless of the type of acid assessed. Of note, the MICs of the tested *E. coli* O26 isolates in the acid challenge assay were comparable to the MIC values observed for the control strain of *E. coli* O157:H7 Sakai with MICs for acetic, citric, propionic and lactic acids of 512, 2048, 1024 and 2048 μg/mL observed.

**Table 5:** MICs_μg/ml_ distribution of acids for 40 *E. coli* O26 isolates from human and cattle

Acids	Tested range (μg/ml)	MIC μg/ml	pH	No of isolates (%)
Acetic acid	64-65563	1024	4.08	35 (87.5)
		512	4.42	5 (12.5)
Citric acid	64-65563	4096	3.31	35 (87.5)
		2048	3.75	5 (12.5)
Lactic acid	64-65563	2048	3.67	40 (100)
Propionic acid	64-65563	1024	4.55	40 (100)

## Discussion

O26 is the second most prevalent serotype identified in cases of foodborne illness attributed to *E. coli* in Australia and throughout the world. A range of measures exist for food producers to limit the spread and transfer of these organisms, however little is known about the variability of response to these control measures by *E. coli* O26 isolates. Isolates included in this study could be categorised into four pathotypes (EHEC, pEHEC, aEPEC and NTEC) based on the presence or absence of EHEC associated markers (*stx*, *eae*, *ehx*). Whilst isolates belonging to the EHEC group are of most interest because of their link to human clinical disease, this study identified a number of pEHEC organisms that appear to differ from EHEC isolates through the absence of *stx*. The ability of EHEC to acquire and lose *stx* has been described previously [38] and consequently there is a need to consider the clinical impact of these isolates.

The development of antimicrobial resistance within *E. coli* and particularly EHEC O26 remains an ongoing concern. In this study, a low level of antimicrobial resistance was observed with 86.4% of isolates susceptible to all antimicrobials tested. These data are consistent with other studies that evaluated the AMR status of *E. coli* in Australian cattle populations at slaughter and in food purchased at retail [39, 40]. These studies determined that approximately >92% of isolates were susceptible to all antimicrobials tested. Furthermore, resistance to antimicrobials of critical or high importance in human medicine was not identified. As previously stated, comparison of the AMR results from different *E. coli* O26 pathotypes determined that although resistance was largely identified in EHEC isolates, these differences were not significant from any of the other pathotypes.

The use of PFGE enabled the identification of two distinct clusters at a similarity level of 75%. Cluster A was primarily composed of EHEC isolates and included a large proportion (98.7%) of isolates that were positive for the *ecf* and SNP within *rmlA* suggesting that these markers are notable features that could be used to define cluster A from cluster B. Interestingly, an individual aEPEC isolate grouped in PFGE cluster A and one EHEC isolate grouped into cluster B where the majority of isolates were aEPEC. This result may reinforce the hypothesis of emergence of EHEC and Non-EHEC by loss and gain of the stx gene. In fact, previous studies showed that conversion of EHEC O26 to *stx*-negative *E. coli* O26 is bidirectional where EHEC O26 lose *stx* genes converting to aEPEC and aEPEC O26 can be lysogenised with Stx-encoding phages to give rise to the emergence of EHEC [38]. Another explanation could be that aEPEC isolates located in the same cluster with EHEC may contain pathogenic O island (OI-122, OI-43. OI-48, OI-50 or OI-57) encoded genes which were found to be significantly associated with aEPEC that showed high similarity to EHEC irrespective of their virulence attributes [41, 42].

*E. coli* O26 strains that have been responsible for a number of foodborne outbreaks or isolated from a variety of food matrices and food producing animals draw attention to their tolerance to the environmental stresses applied in the food processing areas and food industry [19, 21, 25, 43]. Subsequently, an evaluation for the effectiveness of the sanitizers used in the food processing environment, and food contact surfaces is crucial for understanding effective pathogen control. Both Profoam and Kwiksan are Quaternary Ammonium Compound (QACs) cationic surfactants that are widely used in clinical and industrial settings. Similarly, Topactive DES. which has peracetic acid as its active ingredient is used in the food industry and for disinfection of medical supplies. The observed MICs of *E. coli* O26 of different pathotypes to a range of QACs (Profoam and Kwiksan) and Topactive DES demonstrated that the required MICs for inhibiting the growth of *E. coli* O26 carrying EHEC virulence markers (EHEC) and pathotypes lacking EHEC virulence factors (aEPEC, pEHEC and NTEC) are similar to that for *E. coli* O157 Sakai strain used as a control in our study. This suggests that the manufacturers’ recommended concentrations for the tested disinfectants validated for O157 strain are effective for the control of *E. coli* O26 of various pathotypes and are may indeed be effective for most *E. coli* regardless of pathotypes or serogroup.

When challenged for their capability to survive disinfectants with sodium hypochlorite as the main component, both human and cattle *E. coli* O26 isolates of different pathotypes achieved MICs that exceeded the application concentrations. Consequently all forty isolates regardless of their pathotype were deemed to be non-susceptible to the recommended concentrations of Maxifoam and Dairy Chlor 12.5%. Pathogens that survive recommended concentrations of tested disinfectants pose a greater risk of spreading into the food supply chain and subsequently could contribute to the incidence of human disease thereby reinforcing the importance of continued evaluation of disinfectants. Škaloud et al.*,*[31] reported that the MIC of sodium hypochlorite for both STEC O157 and O26 was 0.5% which is lower than the effective concentrations for disinfection of *E. coli* O26 in this study. Although these data may suggest variations among *E. coli* strains in response to disinfectant stress comparison of the current results with others is difficult since the chosen susceptibility method is different. Previous studies raised concerns about the use of disinfectants and developing resistance to antimicrobial agents [32, 36, 44]. These studies suggested that the use of disinfectants may impose selective pressure giving rise to the emergence of cross-resistance and co-resistance for widely used disinfectants and antimicrobial agents. In our study, the percentage of resistance to a range of disinfectants was similar among O26 isolates regardless of their AMR status and no association between the use of disinfectants and development of resistance to antimicrobial agents was found suggesting that the presence of either resistance has not resulted in selection for the other.

Organic acids have been used in foods as preservatives to enhance microbial safety. In addition, acids may be used as interventions in the beef industry to reduce bacterial contamination. Results presented here show that the majority of isolates (87%), regardless of their source and pathotypes, exhibited elevated MICs (≥1024 μg/ml) to the tested acids. A previous study on the influence of organic acids on *E. coli* O157:H7 demonstrated elevated MICs for those acids as well [32]. In addition, the MICs for the *E. coli* O26 isolates in this study occurred at low pH (4.55-3.31). Molina et al., [45] have shown that STEC O26 and other STEC serotypes (O91:H2, O111:H^-^, O145: H^-^, and O157:H7) did not grow when they were exposed to citric acid and acetic acid at a pH of 4.5. Others reported that treatment with lactic acid at a concentration of 4%, reduced non-O157 including *E. coli* O26 by 2.3 log [30]. Findings from the current and previous studies suggest that *E. coli* O26 isolates of different pathotypes utilize a number of acid resistance mechanisms to prevent the lethal effect of acidic stresses. The capacity of *E. coli* strains to withstand acidic environments during passage and growth of these bacteria in the intestinal tracts of cattle and human and in acidic food is an important factor that influence their ability to survive and subsequently cause disease [46].

## Conclusion

In conclusion, *E. coli* O26 isolated in Australia are a genetically diverse group of organisms that belong to a range of pathotypes. The low level of resistance and the absence of AMR to clinically relevant antimicrobials in Australian cattle bacterial isolates are reflective of the comprehensive controls over the use of antimicrobials in food-production animals in Australia. However, the tolerance of EHEC and *stx*-negative *E. coli* O26 pathotypes (i.e., aEPEC, pEHEC and NTEC) to three of the tested disinfectants (Maxifoam﻿, Dairy Chlor 12.5% and Envirosan) and the elevated MICs_μg/ml_ to the acids examined in this study might contribute to bacterial colonisation of food contact surfaces, which may result in product contamination and subsequently foodborne illness. The ability of *E. coli* O26 isolates to survive a stress intervention was not related to a specific pathotype as isolates lacking EHEC associated markers such as *stx* or *eae* persisted at the same proportion as EHEC strains suggesting that other factors affect persistence of *E. coli* O26 strains. Knowledge of the virulence factors and genetic relatedness of *E. coli* O26 may improve our understanding of the capability of *E. coli* O26 to survive stress and subsequently cause human illness. Continuous evaluation of disinfectants and acids for their efficacy in reducing *E. coli* O26 should be conducted by food industries to assist in ensuring *E. coli* O26 is limited in its capacity to persist in food processing environments and contribute to foodborne disease.

## Abbreviations

aEPEC: Enteropathogenic *E. coli*
AMR: Antimicrobial resistance
ATCC: American type culture collection
*bfp*: Bundle forming pilus
CDC: Centers for disease control
CLSI: Clinical and Laboratory Standards Institute
*eae*: *E. coli* attachment and effacing gene
*ecf*: *eae* positive conserved fragments
EHEC: Enterohaemorrhagic *Escherichia coli*
*ehx*: Enterohemolysin
EUCAST: European Committee on Antimicrobial Susceptibility Testing
HC: Hemolytic colitis
HUS: Hemolytic uremic-syndrome
MICs: Minimum inhibitory concentrations
NARMS: National antimicrobial resistance monitoring system
NCTC: National collection of type cultures
NTEC: Non-toxigenic *E. coli*
PCR: Polymerase chain reaction
pEHEC: Potential enterohaemorrhagic *E. coli*
PFGE: Pulse-field gel electrophoresis
QACs: Quaternary ammonium compound
RFLP: Restriction fragment length polymorphism
SNP: Single nucleotide polymorphism
TSA: Tryptone soya agar
USA: United State of America
